# Antioxidant and Anti-Obesity Properties of Acidic and Alkaline Seaweed Extracts Adjusted to Different pH Levels

**DOI:** 10.3390/md23010035

**Published:** 2025-01-12

**Authors:** Sakhi Ghelichi, Mona Hajfathalian, Sara Falcione, Charlotte Jacobsen

**Affiliations:** 1 National Food Institute, Technical University of Denmark, 2800 Kongens Lyngby, Denmark; saghel@food.dtu.dk (S.G.); monhaj@food.dtu.dk (M.H.); 2 Department of Health Technology, Technical University of Denmark, 2800 Kongens Lyngby, Denmark

**Keywords:** extraction, pH adjustment, protein recovery, *Palmaria palmata*, radical scavenging, chelating, anti-obesity

## Abstract

This research examined antioxidant and anti-obesity effects of *Palmaria palmata* extracts obtained through acidic or alkaline treatments and subsequent pH adjustments. After two rounds of acidic or alkaline extraction, the extracts were separated from biomass and adjusted to different pH values: for acidic extracts, pH 3 (no adjustment), pH 6, pH 9, and pH 12; for alkaline extracts, pH 12 (no adjustment), pH 9, pH 6, and pH 3. The findings revealed that extraction medium as well as subsequent pH adjustments significantly influenced composition of the extracts in terms of protein content and recovery, amino acids, and phenolic compounds (*p* < 0.05). Acidic conditions produced extracts with potent radical scavenging, especially at pH 6 (IC_50_ = 0.30 ± 0.04 mg.mL^−1^), while alkaline conditions favored metal chelating, with the highest Fe^2+^ chelation at pH 12 (IC_50_ = 0.65 ± 0.03 mg.mL^−1^). Moreover, extracts showed inhibitory activities against porcine pancreatic lipase and α-amylase, with the acidic extract at pH 9 showing the best anti-obesity properties (IC_50_ = 5.38 ± 0.34 mg.mL^−1^ for lipase and IC_50_ = 5.79 ± 0.30 mg.mL^−1^ for α-amylase). However, the highest α-amylase activity was in the alkaline extract at pH 12 (IC_50_ = 3.05 ± 0.66 mg.mL^−1^). In conclusion, adjusting the pH of seaweed extracts notably influences their bioactive properties, likely due to changes in the reactivity and interactions of bioactive compounds such as peptides, carbohydrates, and polyphenols.

## 1. Introduction

Oxidative stress caused by free radicals can lead to severe health conditions, such as cancer, Alzheimer’s, and neurodegenerative diseases [1]. Natural sources of antioxidant bioactives can mitigate these issues by scavenging free radicals and thereby exerting their antioxidant properties [2]. In addition, oxidation is a significant challenge in the production of foods enriched with n-3 polyunsaturated fatty acids (PUFAs) [3], which are otherwise highly beneficial in preventing severe conditions like cardiovascular diseases, type 2 diabetes, and depression [4]. Oxidative reactions in these enriched foods result in off-flavors, unfavorable textures, and unpleasant odor, and may also cause potential health risks [3]. While synthetic antioxidants have been used to stabilize PUFA-rich foods, they have been associated with health problems in consumers. Therefore, there is a high demand for natural solutions to replace synthetic antioxidants [5].

Obesity, caused by an imbalance between energy intake and expenditure, leads to excess triglycerides in adipose tissues and significantly increases the risk of diabetes, both of which are severe global health issues [6]. Bioactive macromolecules such as peptides [7,8] and polyphenols [9] from natural sources have demonstrated promising potential as anti-obesity and anti-diabetic agents. This highlights the importance of exploring natural compounds for developing effective treatments for these conditions.

Seaweed has gained substantial attention as a source of bioactive compounds with nutritional and medicinal properties [10]. Reasons for this trend include the sustainability of harvest and production, diverse nutritional profile, and suitability for plant-based diets [11]. However, the extraction of biomolecules from seaweed is limited by its complex and rigid cell wall, which hinders the efficiency of extraction methods [12]. Therefore, it is essential to employ efficient procedures to obtain the bioactive compounds from seaweed, as these compounds can mitigate oxidative stress, reduce lipid oxidation, and potentially address obesity-related health issues.

Extraction using acid or alkaline media offers an efficient and economically feasible solution for extracting biomolecules from seaweed [13]. However, the extracts obtained through these methods, or sequential methods such as enzymatic treatments followed by acid or alkaline extraction, are usually highly acidic or alkaline, which may limit the application of the extracts. In this regard, pH changes after extraction while the supernatant is not separated from the treated biomass might lead to a change in or “washout” of otherwise bioactive molecules from the extracts. Therefore, it is interesting to study the properties of these highly acidic or alkaline extracts when their pH is adjusted after separating them from the substrate. This is particularly important because pH has been identified as a crucial factor influencing the properties of seaweed extracts by influencing their biomolecules [13]—for example, by changing surface properties of proteins and peptides [14], protonating [15] or deprotonating [16] phenolic compounds, and modifying carbohydrates [17]. Furthermore, seaweed extracts may contain naturally occurring complexes and conjugates because of interaction among proteins and peptides, polyphenols, and polysaccharides, which have shown potential bioactivities [18]. These interactions have been reported to be affected by pH [19]. Therefore, the present study aimed to analyze the effects of various pH adjustments on antioxidant and anti-obesity properties of extracts obtained from red seaweed *P. palmata* after either acidic or alkaline extractions. To achieve this aim, we adjusted the pH values of acidic and alkaline extracts to 3, 6, 9, and 12 and coded them as Ac-3, Ac-6, Ac-9, and Ac-12 for acidic extracts, and Ak-12, Ak-9, Ak-6, and Ak-3 for alkaline extracts. By exploring both acidic and alkaline extractions and the following pH adjustments, we sought to understand how different pH levels influence these bioactive properties. Our findings will provide insights into the potential applications of these extracts in functional foods and nutraceuticals.

## 2. Results

### 2.1. Protein Content, Protein Recovery, and Degree of Hydrolysis (DH)

The protein content (% dry matter), protein recovery, and washed-out protein of the *P. palmata* extracts obtained after acidic and alkaline extractions and adjusted to different pH values are shown in Table 1. The protein content (% dry matter) of Ak samples were significantly higher than those of Ac samples, as indicated by a *p*-value of less than 0.05 (*p* < 0.05), which demonstrates the statistical significance of this difference. Extracts obtained after alkaline extraction had significantly higher protein recovery than those acquired after acidic extraction (*p* < 0.05). As expected, no significant difference was observed in the protein content and recovery among different samples from either the acidic or alkaline extractions (*p* > 0.05). Although some precipitations were observed in the Ac samples and Ak-3, which contained a considerable amount of protein (~12–18% for Ac samples and ~39% for Ak-3), this washed-out protein did not considerably contribute to the reduction in protein content and recovery. This is because the amount of precipitation was very small (~0.05–0.1 g precipitates compared to ~3.5–7 g extracts).

**Figure 1:**
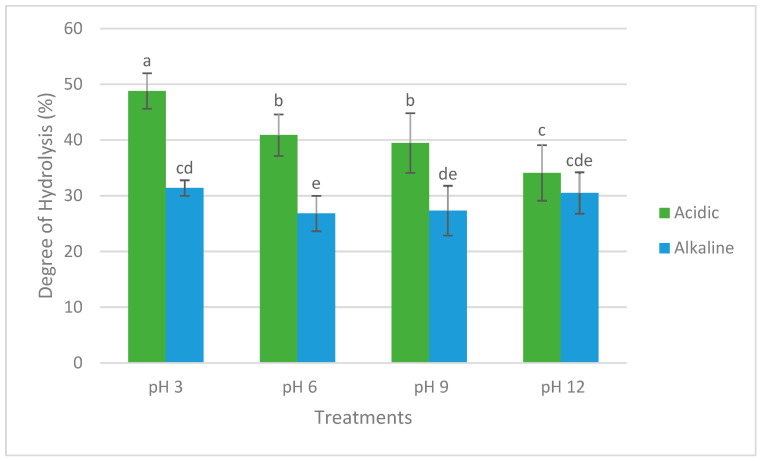
Degree of hydrolysis (DH) of *P. palmata* extracts after acidic and alkaline extractions and subsequent pH adjustments. Data are expressed as mean ± standard deviation (*n* = 16). Different letters denote significant differences among the treatments in terms of DH (*p* < 0.05).

The DH of the seaweed extracts obtained after acidic and alkaline extractions and adjusted to different pH values are shown in Figure 1. A downward trend is observed in the DH of acidic extracts as their pH increases. The highest DHs were observed in the Ac samples, with Ac-3 showing a significantly higher DH than all other samples (*p* < 0.05). Among the Ac samples, no significant difference was observed between Ac-6 and Ac-9 (*p* > 0.05), but the DH in Ac-12 was significantly lower than in the other Ac samples (*p* < 0.05). For the Ak samples, Ak-3 showed the highest DH, which was significantly higher than that of Ak-6 (*p* < 0.05), but not significantly different from Ak-12 and Ak-9 (*p* > 0.05).

### 2.2. Amino Acid Composition

The amino acid analysis results of *P. palmata* extracts after acidic and alkaline extractions followed by pH adjustments are presented in Table 2. The total amino acid (TAA) results aligned with the protein measurement results (% dry matter; Table 1), except for Ak-12, which exhibited significantly lower cystine content compared to other Ak samples (*p* < 0.05). A similar trend was observed in the essential amino acid (EAA) results and the EAA/TAA ratio, where Ak-12 showed significantly lower values compared to other Ak samples (*p* < 0.05). The pH adjustments after acidic extraction resulted in the degradation of tyrosine, histidine, and lysine in the extracts. Except for lysine, Ak-9 had significantly higher content of all amino acids than other treatments (*p* < 0.05), while no significant differences were observed among other Ak samples for most amino acids (*p* > 0.05). Additionally, in all cases except for cystine in Ak-12, Ak samples had significantly higher amino acid contents than Ac samples (*p* < 0.05). Overall, as expected based on the protein content of the extracts (% dry matter), alkaline extraction resulted in a higher amino acid content in the extracts compared to acidic extraction.

### 2.3. Total Phenolic Content

**Figure 2:**
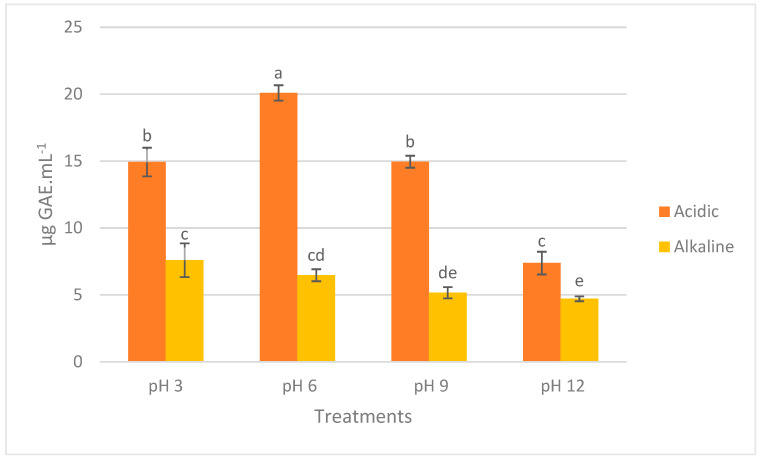
Total phenolic content (TPC) of *P. palmata* extracts after acidic and alkaline extractions and subsequent pH adjustments. Data are expressed as mean ± standard deviation (*n* = 6). Different letters denote significant differences among the treatments in terms of TPC (*p* < 0.05).

The total phenolic content (TPC) of the acidic and alkaline extracts derived from the red seaweed is shown in Figure 2. At first glance, acidic extracts exhibited higher TPC compared to their alkaline counterparts. There is an upward trend in the TPC of alkaline extracts as their pH decreases. Acidic extracts at pH values of 3, 6, and 12 exhibited significantly higher phenolic compound content than other treatments (*p* < 0.05). The highest TPC was observed in Ac-6 (20.10 ± 0.58 µg gallic acid equivalent (GAE).mL^−1^ equaling 0.0201 ± 0.0006 mg GAE.g^−1^), which was significantly higher than in all other treatments (*p* < 0.05). Among alkaline extracts, Ak-3 had the highest TPC (7.60 ± 1.26 µg GAE.mL^−1^ equaling 0.0076 ± 0.0013 mg GAE.g^−1^), showing a significant difference compared to Ak-9 and Ak-12 (*p* < 0.05). In both Ac and Ak samples, pH 12 resulted in a reduction in TPC, which was significantly different in all cases (*p* < 0.05), except between Ak-12 and Ak-9 (*p* > 0.05). At each tested pH value, TPC in acidic extracts was significantly higher than in alkaline extracts (*p* < 0.05).

### 2.4. Antioxidant Properties

Table 3 presents the IC_50_ values for 1,1-diphenyl-2-picrylhydrazyl (DPPH) radical scavenging activity and Fe^2+^ chelating activity of acidic and alkaline extracts from *P. palmata* adjusted to different pH values. The acidic samples at pH values of 3, 6, and 9, as well as Ak-3, exhibited potent radical scavenging activities, as indicated by their IC_50_ values being less than 1. In contrast, the other samples did not show favorable radical scavenging activity, as evidenced by their relatively high IC_50_ values (greater than 6) or their inability to reach the IC_50_ value at the highest concentration tested (8 mg.mL^−1^). The highest radical scavenging activity was observed in Ac-6 (IC_50_ = 0.30 ± 0.04 mg.mL^−1^), which was significantly higher than that of all other samples (*p* < 0.05), except for Ac-9 and Ak-3 (*p* > 0.05).

The highest Fe^2+^ chelating activity was detected in Ak-12 (IC_50_ = 0.65 ± 0.03 mg.mL^−1^), which was significantly better than that in all other samples (*p* < 0.05) except Ac-12 (*p* > 0.05). Neither of the extracts at pH 3 were able to chelate at least 50% of the metal ions, failing to reach the IC_50_ value at the highest concentration tested (8 mg.mL^−1^). Both Ac-12 and Ak-9 also demonstrated relatively favorable Fe^2+^ chelating activities, with IC_50_ values of less than 2. In contrast, the remaining samples were not as effective as metal ion chelators, as indicated by their high IC_50_ values.

### 2.5. Anti-Obesity Properties

The anti-obesity properties of the acidic and alkaline extracts of *P. palmata* adjusted to different pH values after extraction are shown in Table 3 in terms of their capacity to inhibit the metabolic enzymes α-glucosidase, lipase, and α-amylase. None of the extracts, whether obtained through acidic or alkaline extraction methods, could inhibit α-glucosidase.

Acidic extracts demonstrated better lipase inhibition compared to alkaline extracts. The highest lipase inhibition was observed in Ac-9 (IC_50_ = 5.38 ± 0.34 mg.mL^−1^), which was significantly better than all other extracts (*p* < 0.05). Following Ac-9, Ac-6 also showed significantly higher lipase inhibition than other extracts (IC_50_ = 7.10 ± 0.29 mg.mL^−1^) (*p* < 0.05), but its inhibition was significantly lower than that of Ac-9 (*p* < 0.05). Except for these two extracts, none of the others could inhibit 50% of the lipase to reach IC_50_ values at the highest concentration tested (8 mg.mL^−1^). Among alkaline extracts, Ak-6 showed the highest lipase inhibition at the highest concentration tested (34.46 ± 2.40%), which was significantly higher than that of other alkaline extracts (*p* < 0.05). However, none of the alkaline extracts reached the IC_50_ value at the highest concentration tested (8 mg.mL^−1^). No significant difference was detected in lipase inhibition between Ak-6 and Ac-12 (35.03 ± 2.66%), between Ac-3 (24.34 ± 5.31%) and Ak-3 (29.19 ± 2.09%), or between Ak-12 (17.97 ± 1.49%) and Ak-9 (13.51 ± 1.17%) at the highest concentration tested (8 mg.mL^−1^) (*p* > 0.05).

The highest α-amylase inhibition was detected in Ak-12 (IC_50_ = 3.05 ± 0.66 mg.mL^−1^), which had a significant difference from other extracts (*p* < 0.05). However, the other alkaline extracts did not show favorable α-amylase inhibition activity, as none of them reached the IC_50_ values at the highest concentration tested (8 mg.mL^−1^). Ak-9 (31.56 ± 4.03%) and Ak-3 (35.59 ± 4.74%) showed significantly higher α-amylase inhibition than Ak-6 (11.86 ± 2.38%) at 8 mg.mL^−1^ (*p* < 0.05). Among acidic extracts, Ac-6 (IC_50_ = 5.97 ± 0.73 mg.mL^−1^) and Ac-9 (IC_50_ = 5.79 ± 0.30 mg.mL^−1^) exhibited significantly higher α-amylase inhibition than Ac-12 (IC_50_ = 7.39 ± 0.89 mg.mL^−1^) (*p* < 0.05), while Ac-3 inhibited only 27.40 ± 4.15% of the enzyme activity and did not reach the IC_50_ value at the highest concentration tested (8 mg.mL^−1^).

## 3. Discussion

### 3.1. Protein Content, Protein Recovery, and Degree of Hydrolysis

A clear trend observed in the protein content and recovery was the significantly higher levels in extracts obtained after alkaline extraction compared to acidic extraction (*p* < 0.05). This suggests that alkaline solvents may enhance the extraction and release of more protein from the seaweed matrix into the supernatant, resulting in protein-rich extracts. This observation can be understood from various perspectives. One is that the increased protein solubility in alkaline conditions is due to the higher net surface charge of proteins, leading to stronger electrostatic repulsion. Protein solubility is influenced by pH; for instance, proteins are less soluble around their isoelectric point because they lack a net charge and cannot repel each other effectively. However, at pH levels far from the isoelectric point, electrostatic repulsions become stronger, enhancing protein solubility [21]. This study suggests that alkaline conditions may create a more favorable environment for these repulsions compared to acidic conditions, potentially resulting in higher protein solubility and recovery in seaweed extracts. This is further supported by the results of the washed-out protein observed after adjusting the pH following the separation of extracts from the biomass. The findings indicated that in acidic extracts, adjusting the pH towards higher values significantly decreased the amount of protein washed out from the extract (*p* < 0.05). Similarly, in alkaline extracts, adjusting the pH of the extract (after separating from the biomass) to 3 resulted in a sediment containing a considerable amount of protein. However, since the amount of this sediment was negligible, it did not notably affect the protein recovery in the extract. Nonetheless, this should not be considered unimportant, as it could become a concern in large-scale operations, potentially resulting in the loss of a considerable amount of protein.

A second perspective is that alkaline conditions effectively disintegrate the seaweed cell wall, providing better access to the proteins trapped within the cell wall and enhancing their extraction [12]. It should be noted that although acidic conditions can also break down the cell wall and improve access to intracellular proteins, alkaline conditions not only disintegrate the cell wall but also enhance protein solubilization [13]. This may explain why, in the current study, the protein content and recovery from alkaline extractions were higher than those from acidic extractions.

Another possible explanation is the interaction between the released proteins (or resulting peptides after chemical hydrolysis) and other macromolecules in the seaweed matrix, such as polysaccharides. These interactions can form aggregates, leading to protein precipitation and reduced solubility. At lower pH values, protein surface charges may promote interactions with charged polysaccharides (or polyphenols), contributing to the formation of insoluble aggregates [22]. Studies have shown that at pH values below 5, protein–polysaccharide interactions may result in the formation of insoluble complexes [23]. This aligns with the current study’s findings, where acidic extraction resulted in lower protein content and recovery, and adjusting the extract’s pH towards acidic values led to higher protein washout.

Furthermore, alkaline conditions can cause proteins to partially denature and unfold, which exposes their active groups and makes them more accessible for extraction [24]. However, this explanation should be used cautiously, since strong alkaline conditions might result in the formation of lower-molecular-weight fractions. These fractions might associate through hydrophobic interactions and intermolecular disulfide bonds, which leads to the formation of insoluble aggregates [25]. However, protein aggregation and the resulting decrease in solubility may also occur under acidic pH conditions [26]. It is likely that the formation of insoluble aggregates in acidic conditions is so pronounced that it overshadows the occurrence of similar phenomena in alkaline conditions, if they happen at all.

The findings of this study indicate a notable trade-off associated with acidic extraction. While this method resulted in lower protein content and recovery in the extracts, it achieved higher DH. This suggests that acidic conditions are more effective at breaking down protein structures, leading to a greater extent of hydrolysis. The higher DH values observed could be advantageous in applications where smaller peptide fragments are desired, such as in the production of bioactive peptides or in improving peptide stability during gastrointestinal digestion [27]. However, the reduced protein yield might limit the overall efficiency of protein recovery. This could be a critical factor in industrial applications where maximizing protein extraction is essential. In addition, caution is warranted, as alkaline conditions might alter protein conformation, making certain bonds less accessible to the o-phthaldialdehyde (OPA) reagent used for DH measurement in this study [22]. This is further highlighted by a decreasing trend observed in the present study in the DH of acidic extracts as their pH was adjusted to higher values. Another possibility is that higher pH values may involve certain amino acid residues, such as phenylalanine, tyrosine, tryptophan, and histidine, in non-covalent bonds like π–π interactions [28]. This can potentially lead to the self-assembly of the peptides [29], contributing to the lower DH observed in the acidic extracts at higher pH conditions. Overall, when applying acidic or alkaline solvents for protein extraction from seaweed, it is essential to optimize extraction conditions to balance protein recovery and DH, thereby enhancing the practical applications of the resulting extracts.

### 3.2. Amino Acid Composition

Based on the analysis of protein content in both acidic and alkaline extracts, it was anticipated that the alkaline extracts would have a significantly higher amino acid content, which was confirmed by the results of the amino acid analysis. Notably, the alkaline extracts, except for Ak-12, demonstrated a significantly better essential to total amino acid ratio (*p* < 0.05). This suggests that alkaline solvents are more effective than acidic solvents in solubilizing essential amino acids or generating peptides with backbones containing more essential amino acids than non-essential ones. This finding is also important from a nutritional perspective, highlighting the potential benefits of the resulting extracts. The highest amino acid content was observed in Ak-9, which was significantly higher than other treatments in most cases (*p* < 0.05). However, at pH 12, there was a considerable decrease in amino acid content in the alkaline extract. This can be attributed to changes in protein functionality and digestibility under extreme alkaline conditions, including denaturation, cross-linking, and racemization of amino acids; these changes lead to the loss of essential amino acids [21]. Interestingly, lysine was an exception, with its content being significantly higher in Ak-12 compared to other samples (*p* < 0.05). This suggests that the highest pH condition in alkaline extracts plays a role in solubilizing more lysine. Extreme alkaline conditions are known to potentially damage lysine [30]. However, at pH values above the isoelectric point of lysine (around pH 12), the amino acid carries a net negative charge. This causes a strong electric repulsion [31], which may enhance its solubility.

Another observation regarding the amino acid content of the extract in the present study was the presence of cystine, which was detected in all the extracts. Previous studies on the sequential enzymatic/alkaline extraction of protein from *P. palmata* attributed the cystine content in their fractions to N-acetyl cysteine (NAC) being used as a reducing agent during alkaline extraction [20,32], which was also employed in the current study. Another possible reason for the presence of cystine could be the oxidation of cysteine. When two cysteine molecules undergo oxidation, they form a disulfide bond, resulting in cystine [33]. Research has shown that cysteine oxidation does not require enzymatic catalysis; instead, changes in the redox state, leading to the spontaneous protonation or deprotonation of amino acid side chains due to pH changes, can trigger the oxidation [34]. Therefore, in addition to the effect of NAC content in the solvents, the reduction in pH from the extreme value of 12 in the alkaline extracts might have favored the oxidative interaction of the released cysteines from seaweed matrix, contributing to the noticeably higher rates of cystine formation. This could explain the pronounced mismatch between the protein content and the total amino acid content for Ak-12, considering that our method cannot measure cysteine. The cystine content for Ak-12 was found to be around 4 mg.g^−1^ of dry weight, while it ranged from approximately 17 to 24 mg.g^−1^ of dry weight for the other alkaline extracts.

### 3.3. Total Phenolic Content

Extraction using acidic solvent resulted in significantly higher phenolics in the extracts compared to the use of alkaline solvent (*p* < 0.05). A similar observation was reported in a study on the effect of pH during aqueous extraction from seaweed, where lower pH contributed to higher TPC [35]. This phenomenon can be approached from different aspects. Firstly, phenolic compounds are susceptible to oxidative degradation under extreme alkaline extraction conditions, which has been shown to considerably reduce TPC [36]. However, the specific pH values in the alkaline condition should be considered. For instance, pH 9 in another study on *P. palmata* contributed to considerably higher TPC during aqueous extraction [22], while the pH value of approximately 12 in the present study resulted in a notable decrease in TPC compared to the extracts obtained after acidic extraction. Furthermore, acidic conditions might influence the binding of phenolic compounds with proteins and polysaccharides in seaweed in a way that increases the solubility of the phenolics, leading to higher TPC values. Research has shown that lower pH values favor the binding between polyphenols and proteins, which might yield either soluble or insoluble aggregates [37]. In addition, polyphenols can form soluble complexes with cell wall polysaccharides under acidic conditions [38]. Therefore, the choice of pH conditions plays a crucial role in the extraction efficiency of phenolic compounds. For instance, acidic conditions generally enhance the phenolic content in the extracts compared to extreme alkaline conditions. This understanding can guide the optimization of extraction processes for maximum yield of beneficial phenolics from seaweed.

Adjusting the pH of acidic extracts after separating the biomass significantly increased TPC at pH 6 and significantly decreased it at pH 12 (*p* < 0.05). This could be due to the interactive dynamics of proteins with polyphenols at pH values close to and far from their isoelectric points. Studies have shown that the interactions between polyphenols and proteins are greatly affected by pH conditions, particularly in relation to the isoelectric points of the proteins [39,40]. Our results indicated that adjusting the pH of acidic extracts to values close to the seaweed proteins’ isoelectric points resulted in higher TPC, while adjusting their pH to extremely alkaline conditions led to a considerable decrease in TPC. This could be due either to the formation of soluble protein–polyphenol aggregates at the seaweed protein’s isoelectric point or to the dissociation of protein–polyphenol complexes at this point. It is noteworthy that the former seems unlikely since the pH adjustment was performed after the separation of extracts from the biomass. Therefore, the pH change could not increase TPC by forming soluble aggregates with already-existing polyphenols in the extracts. However, the latter hypothesis deserves attention, as the complexation of proteins and polyphenols might lead to the underestimation of phenolic contents in the extracts by interfering with their reaction with the Folin–Ciocalteu reagent used in the TPC analysis. This hypothesis warrants further research.

### 3.4. Antioxidant Properties

The extracts obtained using the acidic solvent were potent radical scavengers, except when the pH was adjusted to a highly alkaline condition (pH 12). These findings align with the results of TPC analysis, where acidic extracts, except the one adjusted to pH 12, had significantly higher TPC than other extracts (*p* < 0.05). Notably, Ac-6, which had a significantly lower IC_50_ value compared to Ac-3 and Ac-9 (*p* < 0.05), also had significantly higher TPC than the other two extracts (*p* < 0.05). This indicates a direct relationship between phenolics in seaweed extracts and their radical scavenging properties. Previous studies have indicated that phenolic compounds in seaweed extracts can serve as electron donors and bind to free radical ions, thereby scavenging them [41,42]. This is further observed in the results of radical scavenging in alkaline extracts. Ak-3, with higher TPC than other alkaline extracts, showed significantly higher radical scavenging activity compared to other alkaline extracts (*p* < 0.05), and had no significant difference with those of acidic extracts (*p* > 0.05). However, since significant differences were detected in TPC of Ak-3 compared with Ac-3, Ac-6, and Ac-9 (*p* < 0.05), other factors may also play a role in the observed potent radical scavenging properties of these extracts. Although studies have shown that seaweed proteins and peptides can also be important radical scavengers in seaweed extracts [43], this deduction cannot be certainly considered in the context of the present study. This is because Ak-3 did not show considerable differences in terms of protein and amino acid contents and DH compared to Ak-9, which could not scavenge 50% of DPPH radicals at the highest concentration tested (8 mg.mL^−1^). Therefore, it may be that the acidic condition in Ak-3 contributed to the interactions of biomolecules in the extract, including peptides, phenolic compounds, and polysaccharides, leading to the formation of complexes or conjugates that could effectively scavenge free radicals. These complexes and conjugates may be lower or absent in the other alkaline extracts, whose pH was kept at alkaline or near-neutral (pH 6). It has been reported that at low pH values below the isoelectric point of proteins, they are positively charged and are absorbed on the surface of anionic polysaccharides through electrostatic attraction [44]. Similarly, at low pH values, the binding sites of proteins are prone to interact with polyphenols through electrostatic interactions [40]. Therefore, these electrostatic dynamics between proteins and other existing macromolecules in the extracts at low pH values may govern the potent radical scavenging activity of the acidic extracts and the alkaline extract adjusted to acidic pH after extraction.

An opposite trend was observed for the Fe^2+^ chelating properties of the extracts. Both acidic and alkaline extracts adjusted to alkaline pH values showed better chelating properties. This indicates that alkaline conditions favor the formation of aggregates that can effectively chelate metal ions. This deduction is further supported by the observation that seaweed extracts obtained using alkaline solvents generally had better metal ion chelating activities than those obtained using acidic solvents adjusted to the same pH condition. Since no clear trend was found in the composition of the extracts with the highest chelating properties compared to other extracts, the role of alkaline pH is crucial for the observed potent chelating properties. Acidic conditions are known to promote non-covalent bindings between proteins and polyphenols through electrostatic interactions. This could result in complexes with potent radical scavenging properties. However, alkaline conditions have been reported to induce covalent bond formation. Under alkaline conditions, semiquinones formed from polyphenol oxidation rearrange into quinones, which can then form covalent crosslinks between proteins and polyphenols. The resulting conjugates may exhibit antioxidant properties [45]. Therefore, it can be deduced that protein–polyphenol interactions under acidic conditions lead to non-covalent bindings that produce complexes with radical scavenging properties. However, interactions under alkaline conditions result in covalent bonds that form protein–polyphenol conjugates with potent metal ion chelating properties. Another possibility is the formation of covalently bound protein–polysaccharide conjugates with metal ion chelating activity in the extracts. Since the seaweed polysaccharides seem to lack reactive carbonyl groups required for the Maillard reaction, the formation of such conjugates through this reaction is unlikely. However, the alkaline conditions might have created a suitable environment for the naturally occurring covalent grafting reactions between seaweed proteins and polysaccharides. These reactions are reported to occur due to the interaction between acyl donors of the polypeptide chain and amino groups of polysaccharides [46].

### 3.5. Anti-Obesity Properties

Fat-digesting enzymes like pancreatic lipase and carbohydrate-digesting enzymes like α-glucosidase and α-amylase are essential for digesting our food. However, their overactivity can increase intestinal fat and glucose absorption, potentially leading to health problems such as obesity and diabetes [6]. Therefore, natural compounds that can reduce the activity of these enzymes are potentially considered anti-obesity agents. The results of this study revealed that neither acidic nor alkaline extracts showed any inhibitory activity against α-glucosidase at the highest concentration tested (8 mg.mL^−1^). Previous studies have reported that brown seaweed extracts can effectively inhibit α-glucosidase due to their phytochemical content, including carotenoids [47], phenolics [48], and flavonoids and terpenoids [49]. However, it seems that acidic and alkaline extracts of *P. palmata* lack adequate levels of these phytochemicals to inhibit α-glucosidase activity. Another possibility is that these phytochemicals may have degraded during extraction using acidic or alkaline solvents. Therefore, future studies should analyze extract obtained from this seaweed through other methods, such as enzymatic treatments, to assess their activity in inhibiting α-glucosidase.

Unlike α-glucosidase, extracts obtained from red seaweed, especially those using acidic solvent, showed inhibitory activity against pancreatic lipase. Since the protein and amino acid contents of acidic extracts were noticeably lower than those of alkaline extracts, the observed lipase inhibitory activity of acidic extracts, particularly at pH 6 and 9, cannot be directly attributed to their protein or amino acid content. Other biomolecules may play a more pronounced role in the observed effect. This is supported by a study on the lipase inhibitory effects of seaweed extract, which highlighted the role of phenolic compounds, especially phlorotannin, and polysaccharides in the inhibition of pancreatic lipase [50]. An interesting observation was that when the pH of acidic extracts was adjusted to 6 and 9, the lipase inhibition properties increased significantly (*p* < 0.05), while extremely acidic and alkaline conditions did not favor lipase inhibition in the extracts. One reason could be the considerably higher TPC in these extracts. However, this should be approached with caution because Ac-3 also contained considerable TPC, and the TPC in Ac-6 was significantly higher than that of Ac-9 (*p* < 0.05), while an opposite trend was observed for these two extracts in terms of pancreatic lipase inhibition. This could be due to certain conditions of solvents used to extract phenolics, such as pH, causing structural changes in the released phenolics, altering their reactivity to digestive enzymes [51]. Another possible explanation could be the effect of pH conditions on the interactions among the biomolecules in seaweed extracts. These interactions could have yielded aggregates that could effectively bind to pancreatic lipase, inhibiting its activity. It seems that no study has elucidated these interactions as affected by pH in seaweed extracts and their contribution to inhibiting metabolic enzymes. This deserves further research, particularly by identifying the molecular composition of extracts and performing structure–activity investigations to analyze the effects of the biomolecules and their interactions in inhibiting pancreatic lipase.

The seaweed extracts showed more potent inhibitory activity against α-amylase than against pancreatic lipase, with Ak-12 and Ac-9 showing close to 60% inhibition of the enzyme. Since no clear trend is observed in the compositions of these extracts, the observed α-amylase inhibition activity is likely rooted in the structural phenomena occurring for the bioactive compounds after the adjustment in the pH of the extracts. For instance, it has been reported that hydroxyl groups of polyphenols may inhibit α-amylase by forming hydrogen bonds with the active sites of α-amylase or through π–π stacking interactions between aromatic rings of polyphenols and the indole ring of tryptophan at the enzyme’s active site [52]. The pH adjustments in the present study may have exposed more hydroxyl groups or aromatic rings of the polyphenols, enhancing their ability to react with and inhibit α-amylase. Moreover, the effects of pH conditions on the structures of carbohydrates in the extract may influence the observed effects on α-amylase inhibition. Research has shown that carbohydrates in seaweed can inhibit α-amylase by affecting its secondary structure, including α-helix, β-sheet, and β-turn formations [53]. It is noteworthy that the best IC_50_ value for α-amylase inhibition in the present study was 3.05 ± 0.66 mg.mL^−1^ for Ak-12. This IC_50_ value is still considerably higher than that of acarbose (IC_50_ = 8.996 ± 0.030 µg.mL^−1^), a clinical agent used to treat hyperglycemia. However, considering the reported adverse effects of acarbose therapy, such as abdominal distention and diarrhea [54], the seaweed extracts, especially Ak-12 and Ac-9, could offer a potential natural solution that is expected to have minimal side effects and additional health benefits. This should be further investigated to confirm these potential benefits and ensure safety.

## 4. Materials and Methods

### 4.1. Seaweed Biomass Preparation

Air-dried *P. palmata* from a batch collected between late autumn and early winter in 2023 from the Faroe Islands coast was bought from a Danish company (DanskTANG, Nykøbing Sj., Denmark). The biomass was freeze-dried using a ScanVac CoolSafe freeze-dryer (LaboGene A/S, Allerød, Denmark) and pulverized to approximately 0.5–1.0 cm particle size using a laboratory mill (KN 295 Knifetec™, Foss A/S, Hillerød, Denmark). The resulting powder was stored in zip-lock plastic bags at −20 °C in dark conditions.

### 4.2. Chemicals and Enzymes

All solvents used were of high-performance liquid chromatography (HPLC) grade and purchased from Lab-Scan (Dublin, Ireland). Amino acid standards were purchased from Sigma-Aldrich (St. Louis, IL, USA). HPLC-grade water was prepared at DTU Food using a Milli-Q^®^ Advantage A10 water deionizing system from Millipore Corporation (Billerica, MA, USA). Butylated hydroxytoluene (BHT), ethylenediaminetetraacetic acid (EDTA), DPPH radical, α-glucosidase from *Saccharomyces cerevisiae*, porcine pancreatic α-amylase, Acarbose^®^, porcine pancreatic lipase, and Orlistat^®^ were obtained from Sigma–Aldrich (Steinheim, Germany). All other chemicals were obtained from Merck (Darmstadt, Germany).

### 4.3. Extraction Procedure

Sixteen flasks, each containing 6 g of biomass powder and 120 mL of solvent (1:20 *w*/*v*), were prepared for eight samples (treatments in duplicate). The solvents used were either 4 g.L^−1^ sodium hydroxide (NaOH) + 1 g.L^−1^ NAC for alkaline extraction or 3.645 g.L^−1^ hydrochloric acid (HCl) + 1 g.L^−1^ NAC for acidic extraction. Each sample underwent two rounds of extraction, each lasting 24 h, on an orbital shaker at 130 rpm at room temperature. After the first round, the suspended biomass was filtered, and the supernatant was collected in separate containers and stored at 4 °C. The biomass was then re-suspended in fresh solvent (alkaline or acidic, as appropriate). Following the second round of extraction, the biomass was filtered and freeze-dried. The supernatants from both rounds were pooled and immediately subjected to pH adjustments. For the acidic extracts, the initial pH was around 3 and was adjusted to 6, 9, and 12 (Ac-3, Ac-6, Ac-9, and Ac-12, respectively). For the alkaline extracts, the initial pH was around 12 and was adjusted to 9, 6, and 3 (Ak-12, Ak-9, Ak-6, and Ak-3, respectively). After pH adjustments, the extracts were centrifuged at 4400× *g* for 15 min at 4 °C (Thermo Scientific™, Sorvall Lynx 4000, Waltham, MA, USA) and then filtered through a sieve with a mesh size of approximately 1 mm. The extracts were pre-frozen at −20 °C for 2 h and then transferred to a −80 °C freezer for 6 h before being freeze-dried. Any precipitate observed after centrifugation and sieving was collected and freeze-dried. The samples were stored in zip-lock plastic bags at −80 °C until analysis. For mass balance calculations, all fractions were weighed using a laboratory balance with a readability of 0.01 g at different stages.

### 4.4. Protein Content and Recovery

To measure the protein content of samples, the total nitrogen content of the samples was determined via the Dumas combustion method using a fully automated rapid MAX N (Elementar Analysensysteme GmbH, Langenselbold, Germany). About 200 mg of samples were fed into the system, and the exact weight was recorded. The protein content was measured by multiplying the nitrogen content by a factor of 5.0 [32].

Protein recovery in the samples was calculated based on the following equation:

$$Protein\;recovery\;in\;fraction\;(\%)=\frac{M_{F}\times P_{F}}{M_{S}\times P_{S}}$$

where *M_F_*, *P_F_*, *M_S_*, and *P_S_* stand for the mass of the sample, the protein percentage of the sample, the mass of the seaweed, and the protein percentage of the seaweed, respectively.

### 4.5. Degree of Hydrolysis (DH)

The OPA assay was employed to determine the DH, following the procedure outlined in [55]. To prepare the OPA reagent, 10 mL of 0.15 M Na_2_CO_3_•10H_2_O were mixed with 10 mL of 0.6 M sodium bicarbonate (NaHCO_3_) and 88 mg of dithiothreitol (DTT). Separately, 80 mg of OPA were dissolved in 2 mL of 96% ethanol and then combined with 10 mL of 1% sodium dodecyl sulfate (SDS). This mixture was added to the DTT solution and diluted to a final volume of 100 mL with distilled water. Samples were diluted to achieve a protein concentration of 0.05–0.25% and then mixed with the OPA reagent in a microplate. Absorbance was measured at 340 nm using an L-serine calibration curve for quantification. The serine equivalent for the samples was determined as described below.

$$Sample\;\left(mg\;Ser.{mL}^{-1}\right)=\frac{\left({Abs}_{sample}-{Abs}_{blank}\right)-intercept}{slope}\times DF$$

*Abs_sample_*, *Abs_blank_*, and DF denote the sample’s absorbance, the blank’s absorbance, and the dilution factor, respectively. *Intercept* and *slope* were acquired from the L-serine calibration curve. DH (%) was then calculated as shown below.

$$DH\;\left(\%\right)=\frac{Sample\;(mg\;Ser.{mL}^{-1})}{P\times 10}\times 100$$

where *P* stands for the protein content in percentage. The measurement of each dilution was performed in duplicate.

### 4.6. Amino Acid Profile

Approximately 30 mg of the dried sample were hydrolyzed with 6 M HCl at 110 °C for 18 h. The hydrolysates were then filtered into 4 mL vials through 0.22 µm cellulose acetate spray filters using 1 mL syringes. Next, 100 µL of the filtered hydrolysates were pipetted into 4 mL vials. The pH was adjusted by gradually adding 1.5 mL of 0.2 M KOH, followed by 1.6 mL of ammonium acetate buffer (100 mM; pH 3.1 adjusted with formic acid) to achieve a dilution factor of 32. The amino acid composition was analyzed using liquid chromatography coupled with mass spectrometry (Agilent 1260 Infinity II Series, LC/MSD Trap, Agilent technologies, Santa Clara, CA, USA) equipped with a BioZen 2.6 µm Glycan, 100 × 2.1 mm (00D-4773-AN) column (Phenomenex, Torrance, CA, USA) connected to a Quadrupole 6120 MS (Agilent Technologies, USA) with an ESI ion source. The settings were as follows: a flow rate of 0.5 mL.min^−1^, a column temperature of 40 °C, a 1 µL injection volume, and a 16 min run time. A gradient of two mobile phases, A (10 mM ammonium formate in acetonitrile) and B (10 mM ammonium formate in MilliQ water), was used with the following program: 0–2 min 0–5% phase B, 2–7 min 5–20% phase B, 7–8 min 20–80% phase B, 12.1 min 0% phase B, and 12.1–16 min 0% phase B. A mixture of amino acid standards containing 17 amino acids (not containing glutamine, tryptophan, or asparagine) was run at five different concentrations to create standard curves. Samples were analyzed, and amino acids were quantitated using MassHunter Quantitative Analysis version 7.0 software. Due to the initial hydrolysis, the method cannot detect glutamine, asparagine, tryptophan, or cysteine. Glutamine is converted to glutamic acid, and asparagine to aspartic acid, while tryptophan and cysteine are destroyed during hydrolysis.

### 4.7. Total Phenolic Content (TPC)

TPC in samples was measured following the method described in [56]. A 100 μL aliquot of each sample was mixed with 0.75 mL of Folin–Ciocalteu reagent (diluted 1:10) and allowed to react at room temperature for 5 min. Then, 0.75 mL of 6% sodium bicarbonate was added, and the mixture was incubated at room temperature for 90 min. The absorbance was recorded at 725 nm using a Shimadzu UV mini 1240 spectrophotometer (Duisburg, Germany). A standard curve was generated using various concentrations of gallic acid, and the total phenolic content was expressed as gallic acid equivalents in µg.mL^−1^.

### 4.8. DPPH Radical Scavenging Activity

DPPH radical scavenging activity was measured following the method described in [57], with modifications using microtiter plates and a multi-plate reader. The samples were dissolved in distilled water to create solutions of varying concentrations. Then, 150 μL of the solution was mixed with 150 μL of 0.1 mM ethanolic DPPH solution and incubated in the dark at room temperature for 30 min. The absorbance was measured at 515 nm using an Eon™ microplate spectrophotometer (BioTek Instruments, Inc., Winooski, VT, USA). For the blank, distilled water was used instead of the sample. The control was prepared with 150 μL of sample and 150 μL of 95% ethanol. All the measurements were performed in triplicate. A BHT solution (0.2 mg.mL^−1^) served as the positive control. The DPPH radical scavenging capacity was calculated as follows:

$$DPPH\;radical\;scavenging\;activity\;(\%)=\left(1-\frac{\left(A_{s}-A_{c}\right)}{A_{b}}\right)\times 100$$

where *A_s_*, *A_c_*, and *A_b_* stand for absorbance of sample, control, and blank, respectively. Furthermore, the sample concentrations (mg protein·mL^−1^) needed to inhibit 50% of DPPH radical activity (IC_50_ values) were determined by drawing dose–response curves.

### 4.9. Fe^2+^ Chelating Activity

The Fe^2+^ chelating activity of the samples was measured following the method described in [58], with modifications using microtiter plates and a multi-plate reader. Samples were dissolved in distilled water to obtain different concentrations. Each solution (200 μL) was mixed with distilled water (270 μL) and ferrous chloride 2 mM (10 μL). The reaction was blocked after 3 min using 20 μL of ferrozine solution 5 mM. The mixture was then shaken vigorously. After 10 min at room temperature, the absorbance was read at 562 nm by an Eon™ microplate spectrophotometer (BioTek Instruments, Inc., Winooski, VT, USA). For the blank, distilled water was used instead of the sample. It is noteworthy that the acids or bases used to adjust the pH of the extracts were not accounted for in the blanks. Sample control was prepared without adding ferrozine. All the measurements were carried out in triplicate. For positive control, 0.06 mM EDTA was used. The metal chelating activity was calculated as follows:

$${Fe}^{2+}\;chelating\;activity\;(\%)=\left(1-\frac{\left(A_{s}-A_{c}\right)}{A_{b}}\right)\times 100$$

where *A_s_*, *A_c_*, and *A_b_* stand for absorbance of sample, control, and blank, respectively. Also, the sample concentrations (mg protein·mL^−1^) needed to chelate 50% of Fe^2+^ (IC_50_ values) were determined by drawing dose–response curves.

### 4.10. α-Glucosidase Inhibition Activity

The α-glucosidase inhibitory properties of the samples were evaluated through a spectrophotometric method. This method measures samples’ ability to inhibit the enzymatic hydrolysis of p-nitrophenyl-α-D-glucopyranoside (pNPG) by α-glucosidase [59]. In brief, 40 µL of samples, acarbose (positive control) (IC_50_ = 0.573 ± 0.011 mg.mL^−1^), or blank solution (buffer instead of sample) were added to a 96-well microtiter plate. Then, 40 µL of α-glucosidase solution (0.5 U.mL^−1^) were added and mixed for 3 min at 25 °C. Next, after 3 min, 20 µL of 5 mM pNPG solution were added, followed by another 3 min of mixing. After 5 min, the reaction was terminated by adding 100 µL of 0.1 M sodium carbonate solution to each well. Absorbance was read at 405 nm using an Eon^TM^ microplate spectrophotometer (BioTek Instruments, Inc., Winooski, VT, USA) after a 10 min incubation. The inhibition percentage of α-glucosidase by the extracts was calculated as follows:

$$\alpha -glucosidase\;inhibition\;(\%)=\left(1-\frac{A_{s}}{A_{b}}\right)\times 100$$

where *A_s_* and *A_b_* represent absorbance of sample and blank, respectively.

### 4.11. Porcine Pancreatic Lipase Inhibition Activity

The samples’ ability to inhibit porcine pancreatic lipase was determined following the method described in [60], with major modifications. Initially, a 50 mM solution of p-nitrophenyl butyrate (pNPB) was prepared in dimethyl sulfoxide (DMSO) and diluted with a phosphate buffer (0.1 M, pH 8) to a final concentration of 5 mM. All solutions, including the enzyme and test samples, were prepared in this phosphate buffer to ensure consistency. For the positive control, a stock solution of orlistat was prepared in DMSO at a concentration of 1 mg/mL and diluted in the same buffer (IC_50_ = 0.917 ± 0.012 µg.mL^−1^). First, 40 µL of the sample solution were combined with 40 µL of porcine pancreatic lipase (prepared in phosphate buffer at 0.5 mg.mL^−1^). This mixture was then incubated at 22 °C for 20 min. Following this initial incubation, 20 µL of the 5 mM pNPB solution were added to the reaction, which was subsequently incubated at 37 °C for 30 min to allow the reaction to proceed. After incubation, the absorbance was measured at 410 nm using an Eon^TM^ microplate spectrophotometer (BioTek Instruments, Inc., Winooski, VT, USA). The inhibition percentage of pancreatic lipase by samples was calculated as follows:

$$Pancreatic\;lipase\;inhibition\;(\%)=\left(1-\frac{A_{s}}{A_{b}}\right)\times 100$$

where *A_s_* and *A_b_* represent absorbance of sample and blank, respectively.

### 4.12. Porcine Pancreatic α-Amylase Inhibition Activity

The porcine pancreatic α-amylase inhibition was assessed following the method described in [61]. In brief, 30 µL of samples in 30 mM Sorensen’s phosphate buffer (pH 7) were combined with 30 µL of α-amylase solution (0.5 mg.mL^−1^) in the same buffer and incubated at 37 °C for 20 min. The starch solution was prepared at 0.5% *w*/*v* in 30 mM Sorensen’s phosphate buffer (pH 7) by stirring and boiling for 10 min. After incubation, 60 µL of the 0.5% starch solution were added to each well. The reaction mixtures were then incubated at 37 °C for 10 min. The reaction was halted by adding 120 µL of dinitrosalicylic acid (DNS) reagent. The DNS reagent was prepared by dissolving 0.25 g of DNS in 5 mL of 2 N NaOH, then diluting to 12 mL with deionized water, adding sodium tartrate (7.5 g), and bringing up the solution to 25 mL. The 96-well plate was incubated at 100 °C for 7 min and then cooled to room temperature. Absorbance was measured at 540 nm. Acarbose was used as positive control (IC_50_ = 8.996 ± 0.030 µg.mL^−1^). The inhibition percentage of pancreatic α-amylase by samples was calculated as follows:

$$Pancreatic\;\alpha -amylase\;inhibition\;(\%)=\left(1-\frac{A_{s}}{A_{b}}\right)\times 100$$

where *A_s_* and *A_b_* represent absorbance of sample and blank, respectively.

### 4.13. Statistical Analysis

The obtained data were analyzed via analysis of variance (ANOVA), and differences between means were determined using the Tukey test. All the statistical operations were performed in OriginPro 2023 (OriginLab Co., Northampton, MA, USA). Differences were considered significant at *p* < 0.05.

## 5. Conclusions

This research provided insight into the effects of acidity and alkalinity of solvents used for the extraction of bioactive compounds from seaweed, as well as the role of the pH of the obtained seaweed extracts in their observed properties. Alkaline extraction contributed to higher protein recovery and a greater content of amino acids in the extracts. The concentration of seaweed extracts influenced their antioxidant and anti-obesity activities, with higher concentrations generally enhancing these effects. Based on the results of this study, the alkaline extract without any pH adjustment can be used as a potent metal ion chelator and a favorable α-amylase inhibitor. In contrast, the acidic extract adjusted to pH 9 can be considered a strong radical scavenger and a favorable anti-obesity agent in terms of pancreatic lipase and α-amylase inhibition. However, structure–activity research is required to elucidate the effect of pH conditions on the antioxidant and anti-obesity properties of bioactive molecules in seaweed extracts. Additionally, the role of interactions between biomolecules in the extracts in governing these properties needs further investigation. It is recommended that future studies include the identification of the biomolecules in the extracts, such as peptides, polysaccharides, and polyphenols, accompanied by in silico analyses such as molecular docking, to accurately predict the dynamics of the observed properties—for instance, in terms of scavenging free radicals and binding to the active sites of metabolic enzymes.

## Figures and Tables

**Table 1:** Protein content (% dry matter), protein recovery (%), and washed-out protein (%) of *P. palmata* extracts after acidic and alkaline extractions and the remaining solid residues.

	Protein (%)	Protein Recovery (%)	Washed-Out Protein (%) After pH Adjustment *
Ac-3	3.55 ± 0.38 ^b^	24.52 ± 1.11 ^b^	17.79 ± 0.79 ^b^
Ac-6	4.11 ± 0.02 ^b^	25.15 ± 0.23 ^b^	14.91 ± 0.43 ^c^
Ac-9	4.15 ± 0.39 ^b^	25.37 ± 2.31 ^b^	12.23 ± 0.84 ^d^
Ac-12	3.50 ± 0.08 ^b^	26.68 ± 0.92 ^b^	11.90 ± 0.06 ^e^
Ak-12	6.88 ± 1.62 ^a^	38.00 ± 1.59 ^a^	-
Ak-9	6.96 ± 0.20 ^a^	37.71 ± 0.61 ^a^	-
Ak-6	6.20 ± 0.49 ^a^	39.80 ± 1.76 ^a^	-
Ak-3	6.10 ± 0.08 ^a^	40.26 ± 0.50 ^a^	38.70 ± 0.72 ^a^

Data are expressed as mean ± standard deviation (*n* = 4). Superscripts denote significant differences among the treatments (*p* < 0.05). Ac-3 to -12: extracts obtained after acidic extraction adjusted to pH values of 3, 6, 9, and 12, respectively; Ak-12 to -3: extracts obtained after alkaline extraction adjusted to pH values of 12, 9, 6, and 3, respectively. * Precipitated protein during centrifugation of extracts subjected to pH adjustments after separation of the biomass.

**Table 2:** Amino acid (mg.g^−1^ of dry weight) contents of *P. palmata* extracts after acidic and alkaline extractions.

	Ac-3	Ac-6	Ac-9	Ac-12	Ak-12	Ak-9	Ak-6	Ak-3
Phenylalanine *	0.26 ± 0.01 ^c^	0.27 ± 0.07 ^c^	0.29 ± 0.01 ^c^	0.26 ± 0.03 ^c^	0.73 ± 0.09 ^b^	0.98 ± 0.02 ^a^	0.76 ± 0.02 ^b^	0.75 ± 0.24 ^b^
Leucine *	0.43 ± 0.01 ^c^	0.43 ± 0.12 ^c^	0.40 ± 0.01 ^c^	0.40 ± 0.07 ^c^	1.39 ± 0.19 ^b^	1.90 ± 0.10 ^a^	1.35 ± 0.05 ^b^	1.37 ± 0.45 ^b^
Isoleucine *	0.26 ± 0.01 ^c^	0.26 ± 0.07 ^c^	0.24 ± 0.01 ^c^	0.26 ± 0.04 ^c^	0.81 ± 0.08 ^b^	1.11 ± 0.06 ^a^	0.84 ± 0.02 ^b^	0.82 ± 0.27 ^b^
Methionine *	0.32 ± 0.12 ^c^	0.02 ± 0.05 ^d^	0.02 ± 0.05 ^d^	ND **	0.55 ± 0.19 ^ab^	0.70 ± 0.18 ^a^	0.42 ± 0.11 ^bc^	0.36 ± 0.05 ^bc^
Tyrosine *	0.18 ± 0.05 ^c^	ND	ND	ND	0.61 ± 0.10 ^b^	0.89 ± 0.14 ^a^	0.53 ± 0.01 ^b^	0.54 ± 0.16 ^b^
Proline	1.84 ± 0.02 ^de^	1.79 ± 0.19 ^e^	1.95 ± 0.03 ^cde^	1.73 ± 0.19 ^e^	2.11 ± 0.20 ^cd^	2.96 ± 0.10 ^a^	2.19 ± 0.06 ^c^	2.61 ± 0.25 ^b^
Valine *	1.09 ± 0.23 ^c^	0.76 ± 0.16 ^c^	0.74 ± 0.05 ^c^	0.68 ± 0.12 ^c^	2.16 ± 0.49 ^b^	2.73 ± 0.17 ^a^	2.08 ± 0.17 ^b^	2.19 ± 0.43 ^b^
Alanine	1.60 ± 0.16 ^c^	1.42 ± 0.15 ^c^	1.52 ± 0.04 ^c^	1.53 ± 0.15 ^c^	2.67 ± 0.28 ^b^	3.71 ± 0.20 ^a^	2.84 ± 0.45 ^b^	3.08 ± 0.67 ^b^
Threonine *	0.73 ± 0.19 ^d^	0.51 ± 0.14 ^de^	0.55 ± 0.08 ^de^	0.41 ± 0.08 ^e^	1.04 ± 0.14 ^c^	1.78 ± 0.18 ^a^	1.37 ± 0.19 ^b^	1.20 ± 0.18 ^bc^
Glycine	1.58 ± 0.21 ^c^	1.02 ± 0.20 ^cd^	0.98 ± 0.11 ^cd^	0.82 ± 0.15 ^d^	2.61 ± 0.42 ^b^	3.38 ± 0.33 ^a^	2.61 ± 0.13 ^b^	3.10 ± 0.69 ^ab^
Serine	1.09 ± 0.27 ^c^	0.59 ± 0.20 ^c^	0.57 ± 0.11 ^c^	0.63 ± 0.15 ^c^	2.36 ± 0.40 ^b^	3.34 ± 0.33 ^a^	2.59 ± 0.20 ^b^	2.86 ± 0.66 ^ab^
Arginine	0.43 ± 0.04 ^c^	0.34 ± 0.10 ^c^	0.33 ± 0.01 ^c^	0.32 ± 0.04 ^c^	0.82 ± 0.09 ^b^	1.25 ± 0.03 ^a^	1.00 ± 0.13 ^b^	0.88 ± 0.29 ^b^
Histidine *	0.24 ± 0.17 ^ab^	ND	ND	ND	0.20 ± 0.14 ^ab^	0.38 ± 0.11 ^a^	0.11 ± 0.05 ^bc^	0.18 ± 0.10 ^b^
Lysine *	3.07 ± 0.75 ^b^	ND	ND	ND	4.39 ± 1.15 ^a^	3.11 ± 0.68 ^b^	2.77 ± 0.48 ^b^	2.99 ± 0.57 ^b^
Glutamic acid	6.18 ± 0.35 ^c^	5.69 ± 0.46 ^c^	6.12 ± 0.18 ^c^	5.61 ± 0.60 ^c^	7.09 ± 0.57 ^b^	9.70 ± 0.20 ^a^	7.14 ± 0.19 ^b^	8.92 ± 0.86 ^a^
Cystine *	5.57 ± 0.61 ^d^	5.19 ± 0.80 ^d^	8.69 ± 1.41 ^c^	5.53 ± 1.18 ^d^	4.27 ± 0.71 ^d^	24.73 ± 2.15 ^a^	17.41 ± 1.59 ^b^	23.08 ± 2.25 ^a^
Aspartic acid	6.25 ± 0.70 ^c^	5.37 ± 0.62 ^c^	6.08 ± 0.63 ^c^	5.51 ± 0.69 ^c^	8.35 ± 0.80 ^b^	10.02 ± 0.77 ^a^	7.86 ± 0.30 ^b^	8.90 ± 0.82 ^ab^
TAA ***	31.12 ± 1.92 ^e^	23.66 ± 2.54 ^f^	28.49 ± 1.91 ^ef^	23.70 ± 2.48 ^f^	42.16 ± 5.18 ^d^	72.65 ± 3.48 ^a^	53.88 ± 1.10 ^c^	63.82 ± 5.34 ^b^
EAA	12.14 ± 1.17 ^de^	7.45 ± 1.04 ^f^	10.92 ± 1.36 ^ef^	7.54 ± 1.03 ^f^	16.13 ± 2.73 ^d^	39.75 ± 4.74 ^a^	27.66 ± 1.04 ^c^	33.48 ± 2.10 ^b^
EAA/TAA	0.39 ± 0.02 ^b^	0.31 ± 0.02 ^c^	0.38 ± 0.02 ^b^	0.32 ± 0.02 ^c^	0.38 ± 0.02 ^b^	0.52 ± 0.01 ^a^	0.52 ± 0.02 ^a^	0.53 ± 0.03 ^a^

Data are expressed as mean ± standard deviation (*n* = 6). Superscripts denote significant differences among the treatments (*p* < 0.05). Ac-3 to -12: extracts obtained after acidic extraction adjusted to pH values of 3, 6, 9, and 12, respectively; Ak-12 to -3: extracts obtained after alkaline extraction adjusted to pH values of 12, 9, 6, and 3, respectively. * Essential amino acids (EAAs) in human nutrition [20]. ** Not detected. *** Total amino acids.

**Table 3:** Antioxidant and anti-obesity properties of *P. palmata* extracts after acidic and alkaline extractions.

	IC_50_ (mg.mL^−1^) for DPPH Radical Scavenging	IC_50_ (mg.mL^−1^) for Fe^2+^ Chelating	IC_50_ (mg.mL^−1^) for α-Glucosidase Inhibition	IC_50_ (mg.mL^−1^) for Pancreatic Lipase Inhibition	IC_50_ (mg.mL^−1^) for α-Amylase Inhibition
Ac-3	0.72 ± 0.04 ^b^	NR *	NR	NR	NR
Ac-6	0.30 ± 0.04 ^a^	6.82 ± 0.76 ^d^	NR	7.10 ± 0.29 ^b^	5.97 ± 0.73 ^b^
Ac-9	0.55 ± 0.03 ^ab^	4.13 ± 0.36 ^c^	NR	5.38 ± 0.34 ^a^	5.79 ± 0.30 ^b^
Ac-12	6.49 ± 0.32 ^c^	1.10 ± 0.23 ^ab^	NR	NR	7.39 ± 0.89 ^c^
Ak-12	NR	0.65 ± 0.03 ^a^	NR	NR	3.05 ± 0.66 ^a^
Ak-9	NR	1.64 ± 0.24 ^b^	NR	NR	NR
Ak-6	6.38 ± 0.34 ^c^	3.43 ± 0.95 ^c^	NR	NR	NR
Ak-3	0.58 ± 0.02 ^ab^	NR	NR	NR	NR

Data are expressed as mean ± standard deviation (*n* = 6). Superscripts denote significant differences among the treatments (*p* < 0.05). Ac-3 to -12: extracts obtained after acidic extraction adjusted to pH values of 3, 6, 9, and 12, respectively; Ak-12 to -3: extracts obtained after alkaline extraction adjusted to pH values of 12, 9, 6, and 3, respectively. * Not reached (the sample could not scavenge at least 50% of DPPH radical, chelate at least 50% of Fe^2+^ ions, or inhibit at least 50% of the metabolic enzymes at the maximum concentration tested, i.e., 8 mg.mL^−1^).

## Data Availability

The data acquired in this study can be obtained at request.
